# Supplementary figures and images for: Impact of the 18F-FDG-PET/MRI on Metastatic Staging in Patients with Hepatocellular Carcinoma: Initial Results from 104 Patients
Source: J Clin Med. 2021 Sep 6;10(17):4017. doi: 10.3390/jcm10174017 (PMC8432497; doi:10.3390/jcm10174017)

Supplementary Figure S1: WB-PET/MRI imaging protocol.

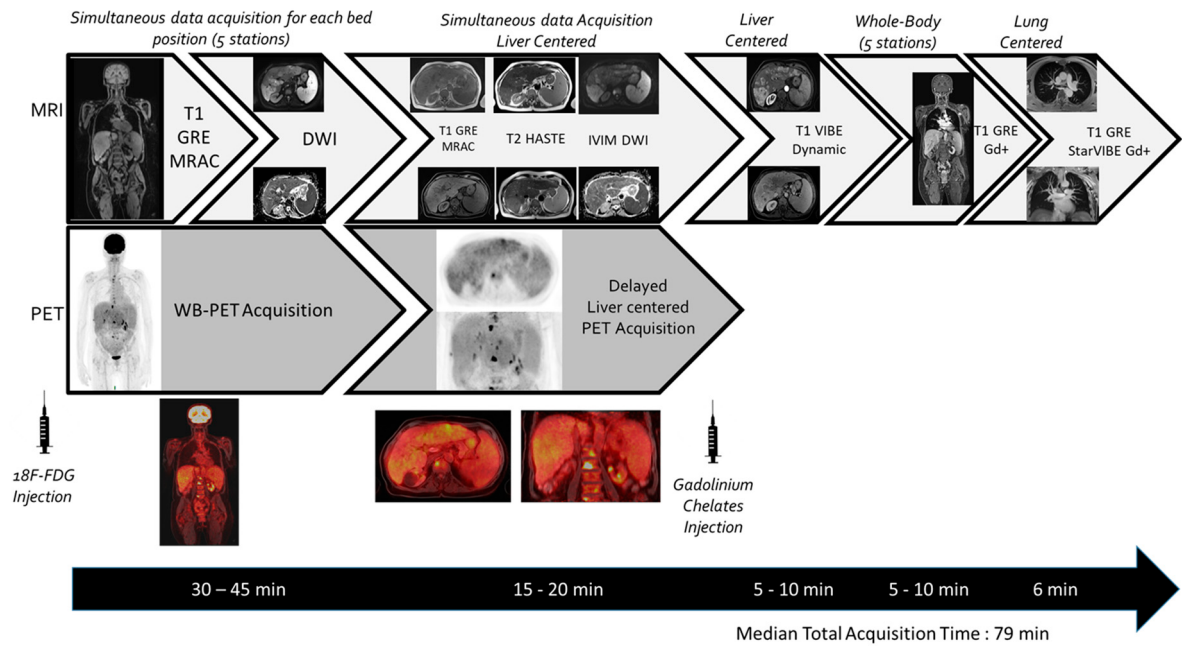

Supplement: Supplementary file 1 [file jcm-10-04017-s001.zip › jcm-1327346-supplementary.pdf]
